# Modeling the Properties of White Matter Tracts Using Diffusion Tensor Imaging to Characterize Patterns of Injury in Aging and Neurodegenerative Disease

**DOI:** 10.3389/fnagi.2022.787516

**Published:** 2022-04-27

**Authors:** Chun Yen Kok, Christine Lock, Ting Yao Ang, Nicole C. Keong

**Affiliations:** ^1^Duke-National University of Singapore (NUS) Medical School, Singapore, Singapore; ^2^Department of Neurosurgery, National Neuroscience Institute, Singapore, Singapore

**Keywords:** diffusion tensor imaging (DTI), white matter, region of interest (ROI), tractography, Alzheimer’s disease, ventriculomegaly

## Abstract

Diffusion tensor imaging (DTI) is a relatively novel magnetic resonance-based imaging methodology that can provide valuable insight into the microstructure of white matter tracts of the brain. In this paper, we evaluated the reliability and reproducibility of deriving a semi-automated pseudo-atlas DTI tractography method vs. standard atlas-based analysis alternatives, for use in clinical cohorts with neurodegeneration and ventriculomegaly. We showed that the semi-automated pseudo-atlas DTI tractography method was reliable and reproducible across different cohorts, generating 97.7% of all tracts. However, DTI metrics obtained from both methods were significantly different across the majority of cohorts and white matter tracts (*p* < 0.001). Despite this, we showed that both methods produced patterns of white matter injury that are consistent with findings reported in the literature and with DTI profiles generated from these methodologies. Scatter plots comparing DTI metrics obtained from each methodology showed that the pseudo-atlas method produced metrics that implied a more preserved neural structure compared to its counterpart. When comparing DTI metrics against a measure of ventriculomegaly (i.e., Evans’ Index), we showed that the standard atlas-based method was able to detect decreasing white matter integrity with increasing ventriculomegaly, while in contrast, metrics obtained using the pseudo-atlas method were sensitive for stretch or compression in the posterior limb of the internal capsule. Additionally, both methods were able to show an increase in white matter disruption with increasing ventriculomegaly, with the pseudo-atlas method showing less variability and more specificity to changes in white matter tracts near to the ventricles. In this study, we found that there was no true gold-standard for DTI methodologies or atlases. Whilst there was no congruence between absolute values from DTI metrics, differing DTI methodologies were still valid but must be appreciated to be variably sensitive to different changes within white matter injury occurring concurrently. By combining both atlas and pseudo-atlas based methodologies with DTI profiles, it was possible to navigate past such challenges to describe white matter injury changes in the context of confounders, such as neurodegenerative disease and ventricular enlargement, with transparency and consistency.

## Introduction

Diffusion tensor imaging (DTI) is a relatively novel magnetic resonance-based imaging methodology that maps the water diffusion properties within the brain (Mori and Zhang, 2006). Since water generally diffuses along intact white matter tracts of the brain, the diffusion properties can therefore provide information about the microarchitecture of specific white matter tracts in the brain. DTI metrics that can be obtained consist of fractional anisotropy (FA), mean diffusivity (MD), axial diffusivity (L1) and radial diffusivity (L2 and 3).

DTI has been used to investigate patterns of white matter changes at a microstructural level in various cohorts, such as normal pressure hydrocephalus (NPH), optic nerve decompression and in the developing human brain (Lebel et al., 2008; Paul et al., 2014; Keong et al., 2017). Diffusion tensor metrics have been shown to be reliable biomarkers for Alzheimer’s disease progression (Acosta-Cabronero et al., 2012), and are also sensitive to changes in white matter injury and compression in patients with NPH after surgical intervention (Scheel et al., 2012; Keong et al., 2017).

However, DTI acquisition, processing, and analysis is a complex multi-step process that is subject to many variables which may affect the results and interpretation thereof (Mukherjee et al., 2008; Soares et al., 2013; Christidi et al., 2016). The post-processing and analysis of DTI metrics is non-trivial and dependent on the availability of software and infrastructure. Quantitative DTI metrics can be obtained by various methods including tract-based spatial statistics (TBSS) which is a voxel-based morphometry-like approach; or the manual placement of 2D region of interest (ROI); atlas-registration based parcellations using a pre-defined white matter atlas to describe tracts of interest (Smith et al., 2006; Mukherjee et al., 2008; Oishi et al., 2009; Soares et al., 2013). or per-image automated tractography approaches, such as TRACULA (Yendiki et al., 2011). ROI analyses are time consuming, influenced by inter-rater variability, and subject to variations along a tract. The Alzheimer’s Disease Neuroimaging Initiative (ADNI) group have previously published (Nir et al., 2013) on the use of both (i) white matter tract atlas ROIs, i.e., registration of images from a DTI atlas to each subject’s distortion corrected FA image, before applying an atlas of white matter labels and superimposing these atlas ROIs into the same coordinate space as subject results for analysis and (ii) TBSS tract atlas ROIs as per (Smith et al., 2006). Per-image automated DTI tractography approaches are an attractive method for disease specific cohorts but are dependent on accurate registration and may be confounded by anatomical differences attributed to neurodegenerative diseases like Alzheimer’s disease and the distortions of white matter tracts secondary to the presence of significant ventriculomegaly, such as in NPH (Mukherjee et al., 2008; Zalesky, 2011; Scheel et al., 2012; Acosta-Cabronero and Nestor, 2014).

In this paper, we evaluated the reliability and reproducibility of differing automated DTI tractography methods to produce diffusion metrics of various white matter tracts in the presence of known confounders such as atrophy in aging, neurodegeneration and significant ventriculomegaly. We firstly aimed to develop a cohort-specific pseudo atlas-based semi-automated tractography method that was comparable to an atlas-based DTI analysis currently utilized by the ADNI group; as we interrogated ADNI datasets for this study, we have therefore defined the latter method as the “gold-standard” approach for reference. We found that the diffusion metrics generated from the former were significantly different from those generated by the latter.

We hypothesized that the results from differing DTI methodologies could be subject to the impact of different algorithmic modifications. To test our hypotheses, we designed the following experiments to optimize the application of DTI methodologies to describe white matter injury patterns in the presence of confounders such as neurodegenerative disease and degree of ventriculomegaly:

1. We developed a cohort-specific pseudo atlas-based semi-automated tractography to generate white matter tracts of interest and compared it to that of the “gold standard” atlas-based DTI analysis currently utilized by ADNI, in order to assess the reproducibility and reliability of the novel methodology.
2. We performed initial comparisons on this pilot to examine the agreement of DTI metrics obtained from white matter tracts generated by both methodologies.
  a. Due to the poor agreement of the metrics, we proceeded to test the differing DTI methodologies under different processing algorithms to assess how these impacted the agreement of the metrics generated.
3. We performed testing using a known model of white matter at-risk of injury. This ROI model allowed us to test for white matter distortion patterns in three cohorts of patients with different levels of confounders, namely varying degrees of neurodegeneration and atrophy along the spectrum from cognitively normal to Alzheimer’s disease.
4. In addition, we performed testing to examine the effect of increasing ventriculomegaly on this ROI model of white matter at-risk. We performed independent quantification of ventricular size by measuring the Evans’ index (EI) and concurrently examined changes in DTI metrics in the context of increasing ventriculomegaly for the ROI model of white matter tracts at risk.
5. Finally, we performed a further layer of analysis to confirm the diffusivity changes in this study by generating morphological patterns of DTI metrics for independent interpretation. We utilized DTI profiles, radar graphs of all DTI metrics (FA, MD, L1, L2, and 3), in order to illustrate differences between DTI methodologies and across cohorts. The concept and utility of DTI profiles has been previously described by our group in describing patterns of white matter injury across clinical cohorts (Lock et al., 2018).

## Materials and Methods

Data used in the preparation of this article were obtained from the ADNI database.^1^ ADNI DTI metrics used for comparison were the UCLA DTI ROI summary measures for ADNIGO and ADNI2.

### Subjects

The ADNI study recruited patients between the ages of 55 and 90 from 57 sites in the United States and Canada. For this study, we retrospectively selected subjects who had screening/baseline MRI scans with diffusion-weighted images (DWI) from the ADNI image data archive. The selected scans included 51 cognitively normal (CN) subjects (mean age 72.47 ± 6.13 years; 45.1% male), 48 patients with Alzheimer’s disease (AD) (75.00 ± 8.67 years; 58.3% male) and 70 patients with early mild cognitive impairment (EMCI) (72.71 ± 8.29 years; 61.4% male). These cohorts were selected because we wanted to interrogate the methodologies in patients with varying degrees of cognitive impairment and atrophy.

### Open-Source Software Used

***3D slicer*** is an open source quantitative imaging network tool we used to derive ventricular morphological indices and to conduct 3D volumetric segmentation (Fedorov et al., 2012). ***MRIcroGL*** is an open source software developed by Neuroimaging Tools and Resources Collaboratory (NITRC) used in this study to convert DICOM images to NIfTI format [(Nitrc) N.T.R.C, 2014]. ***ExploreDTI*** is a graphical toolbox written in MATLAB that was used in this project for DTI and white matter tractography (Leemans et al., 2009).

### MRI Acquisition and Post-processing

MRI scans were performed on 3T GE Medical Systems scanners across participating ADNI sites. Diffusion scans were acquired with 256 × 256 matrix; voxel size 2.7 mm × 2.7 mm × 2.7 mm; 41 DWI (*b* = 1,000 s/mm2) and 5 b0 images. More information on the MRI protocol is available at http://adni.loni.usc.edu/methods/documents/mri-protocols/.

Pre-processing was required to convert each patient’s set of unsorted DICOM format axial DWI images where two-dimensional DICOM image slices were converted into a single 3D NIfTI file with ***MRIcroGL***. During this step, 1 subject in the CN cohort was excluded due to a failure to convert it from DICOM to NIfTI format.

DTI files were generated using ***ExploreDTI***. Thereafter, they were corrected for subject motion and eddy current induced geometric distortion. Whole brain tractography was then performed.

### White Matter Tracts

Utilizing the known ROI model of white matter at-risk, we chose to analyze 8 unique white matter tracts. Bilaterally, we analyzed a total of 14 white matter tracts, and they were as follows: Body of the corpus callosum (bCC), Genu of the corpus callosum (gCC), Inferior fronto-occipital Fasciculus (IFO), Inferior Longitudinal Fasciculus (ILF), Anterior Thalamic Radiation (ATR), Posterior Thalamic Radiation (PTR), Posterior Limb of the Internal Capsule (PLIC), and Uncinate Fasciculus (UF) (Hofer and Frahm, 2006; Wakana et al., 2007; Oishi et al., 2010; Borden et al., 2015; Keong et al., 2017). The bCC and gCC are midline structures while the rest are found bilaterally. Therefore, with 48 DWI in the AD cohort and 50 DWI in the CN cohort, this amounted to a total of 672 and 700 white matter tracts in the AD and CN cohort, respectively.

### Methods of Automated Diffusion Tensor Imaging Tractography

In this paper, two methods of automated tractography were compared. The first method was a cohort-specific pseudo atlas-based semi-automated tractography method (termed **Method 1**) where a randomly selected image in each cohort is used as a template for white matter tract generation in the remaining images. The second method was an automated atlas-based ROI analysis (termed **Method 2**) where a standardized lab-based atlas was used as the template. These two methods were tested on the AD and CN cohorts. We followed this up by implementing additional algorithmic modifications to assess if they affected the results of the methodologies.

We implemented two modifications to the processing algorithms. The first was to try an alternative standardized atlas as a template in Method 2 (using the alternative atlas template is termed **Method 3**). The second was to optimize the alignment to the ACPC plane prior to performing the DTI analysis by following protocol adapted from the Human Connectome Project (HCP) pre-processing pipelines (Glasser et al., 2013). This was done by co-registration of the DWI to the MNI template (standardized template from 152 subject scans) and the corresponding T1-weighted image. This allowed all images in the dataset to be oriented and aligned to the same space such that the anterior and posterior commissures (ACPC) were aligned along a horizontal plane. By ensuring that all images in the dataset were standardized in terms of position and orientation, we sought to improve the fit of both atlases (and the pseudo-atlas) as applied to the images in the dataset. Once we compared and found the technical considerations to have improved the tractography, we subsequently applied the refined methodology to all available cohorts to complete our DTI analysis, with the exception of the EMCI cohort, where only the ACPC alignment was enacted. This was because earlier results from AD and CN cohorts already showed that ACPC alignment improved the tract analysis success rate but did not fully eliminate the large differences between methodologies, so we proceeded to refine our analysis by only using ACPC aligned EMCI scans.

#### Method 1: Automated Atlas Based Tractography

We randomly chose a representative subject in each cohort and set its FA map as a “pseudo-atlas.” To ensure that it was suitable as a template, the image was subject to visual inspection as a quality check and compared to other images to ensure that there were no obvious defects and distortions. Using ExploreDTI, specific white matter tracts of the pseudo-atlas were generated from user-determined regions of interests (ROIs). The type of ROI placed enforced different conditions within the area enclosed by the ROI. Placing an AND ROI generated tracts that passed through this area. Placing a NOT ROI excluded fibers that passed through this area. Table 1 Shows the types of ROIs and their respective locations which were used to isolate the corresponding white matter tracts.

**TABLE 1:** ROI constraints used to isolate white matter tracts in the pseudo-atlas for Method 1.

Tract	ROI constraints
gCC	- Sagittal AND: Define anterior 1/6 of the length of the corpus callosum. - Parasagittal NOT: Slices lateral to corticospinal tract bilaterally, defining entire slice.
bCC	- Splenium of corpus callosum consists of the posterior 1/4 of the length of corpus callosum. - Sagittal AND: Define remaining length of the corpus callosum excluding the genu and splenium—from 1/6 to 3/4 length of corpus callosum. - Axial NOT: Slice just beneath the bCC, defining entire slice.
ATR	- Coronal AND: Slice chosen in the middle of the gCC, defining anterior limb of internal capsule. - Coronal AND: Slice at the anterior edge of pons, defining entire thalamus. - Sagittal NOT: Defining entire central slice. - Coronal NOT: Slice at the posterior thalamic edge, defining entire slice.
IFO	- Coronal AND: Slice at the anterior edge of gCC, defining entire slice. - Coronal AND: Slice at the halfway mark of parieto-occipital sulcus, defining the occipital lobe. - Sagittal NOT: Define entire central slice.
ILF	- Coronal AND: Slice at the posterior edge of cingulum, defining occipital lobe. - Coronal AND: Most posterior coronal slice in which the temporal lobe is not connected to the frontal lobe (as seen on axial view), defining the anterior temporal lobe. - Coronal NOT: Same slice as above, defining the rest of the brain except anterior temporal lobe. - Sagittal NOT: Defining entire central slice.
PLIC	- Axial AND: Slice where PLIC is visibly the largest, defining the PLIC. - Axial AND: Slice at the inferior slice where the PLIC is still visible, defining the PLIC. - Axial NOT: Slice at the condensed portion of the corticospinal tract in the brain stem, defining entire slice.
PTR	- Coronal AND: Slice at the posterior edge of the cingulum, defining anterior-posterior directing, periventricular white matter tracts. - Parasagittal AND: Slice at the lateral edge of thalamus, defining entire thalamus. - Coronal NOT: Slice at the anterior edge of thalamus, defining entire slice. - Axial NOT: slice at the inferior edge of thalamus, defining entire slice.
UF	- Axial AND: Slice where condensed cephalic-caudal directed fibers are distinct in the temporal lobe, defining temporal lobe. - Coronal AND: Slice anterior to the condensed cephalic-caudal directed fibers, defining inferior frontal lobe. - Coronal AND: Same slice as above, defining temporal lobe - Coronal NOT: Slice posterior to the condensed cephalic-caudal directed fibers, defining entire slice.

*gCC, genu of the corpus callosum; bCC, body of the corpus callosum; ATR, anterior thalamic radiation; IFO, inferior fronto-occipital fasciculus; ILF, inferior longitudinal fasciculus; PLIC, posterior limb of the internal capsule; PTR, posterior thalamic radiation; UF, uncinate fasciculus.*

With the pseudo-atlas and ROIs as input, the software applied similar ROIs to the remaining images in the cohort using a deterministic streamline method (Lebel et al., 2008). White matter tracts were then automatically reconstructed via the automatically generated ROIs in the remaining images of the cohort. Where the ROIs extruded to an image failed to generate any tracts, this resulted in missing data.

#### Methods 2 and 3: Automated Atlas-Based Region of Interest Analysis

A widely used standardized DTI template with its associated white matter tracts was used as an atlas. The white matter tracts in the atlas were generated from ROIs determined by the template creator. The atlas template was warped, and the associated ROIs transformed and applied to each image in the dataset. The diffusion metrics were then automatically generated from the resulting white matter tracts defined by the ROIs. The problem of missing data as in Method 1 was also found to occur using this method but was not as significant as in Method 1.

Method 2 utilized the ICBM-DTI-81 atlas from the ICBM DTI workgroup (Oishi et al., 2008). This atlas template was created by averaging hand segmentation of diffusion tensor maps from 81 subjects with a mean age of 39 with 42 males and 39 females.

Method 3 utilized the JHU white matter tractography atlas from the Laboratory of Brain Anatomical MRI, Johns Hopkins University (Oishi et al., 2009). This atlas was based on averaging results from running deterministic tractography on 28 normal subjects with a mean age of 29 with 17 males and 11 females.

The atlas used in Method 2 contained all 8 unique white matter tracts we wanted to investigate whereas the atlas used in Method 3 only identified 5 of the 8 tracts we required: gCC, ATR, IFO, ILF, and UF.

### Characterizing Ventriculomegaly—Evans’ Index

The Evans’ index (EI) is commonly used to characterize the degree of ventriculomegaly from a patient’s MRI or CT scan (Yamada et al., 2016). It is defined as the ratio of the maximum width of the frontal horns of the lateral ventricles to the maximum internal width of the cranial vault as seen on the axial view (Yamada et al., 2016). 3D Slicer was used to re-align T1 scans to the ACPC for consistency and derive the EI (Soon et al., 2021).

### Statistical Analysis

Diffusion metrics FA and MD from both left and right tracts were averaged for the purposes of the analysis. Paired *t*-tests were used to test for differences between the different methodologies. Linear correlation was used to investigate the association between diffusion metrics and ventriculomegaly measured by the Evans’ index. Scatter plots of FA and MD obtained from both methodologies for all tracts across the AD, EMCI, and CN cohorts were plotted to show the agreement of metrics and the variance within each methodology. All statistical analyses were performed using R statistical software (version 4.0.4) (R Development Core Team, 2010). A *p*-value of < 0.05 was considered to be statistically significant.

### Diffusion Tensor Imaging Profiles

DTI profiles are presented as radar graphs of means of all DTI metrics (FA, MD, L1, L2, and 3), in order to provide a simplistic illustration of differences between the various methods, as well as differences across the spectrum of disease for AD. We have previously demonstrated the utility of DTI profiles to describe and compare disease processes in white matter tracts across different cohorts (Keong et al., 2017; Lock et al., 2018). White matter tract profiles were also generated using Tract Analysis Profiles to illustrate how DTI metrics vary **along** each white matter tract (Yeatman et al., 2012).

## Results

### Reproducibility and Reliability of Methodologies

Method 1 generated 96.3% (647/672) of all white matter tracts in the AD cohort and 96.9% (678/700) in the CN cohort. Method 1 was unable to generate 25 tracts in the AD cohort and 22 tracts in the CN cohort. This is in contrast to Method 2 which was able to generate 99.6% (669/672) and 100% (700/700) tracts in the AD and CN cohorts, respectively. This amounts to 3 missing tracts in the AD cohort. After the implementation of the ACPC alignment, the reliability and reproducibility of Method 1 improved with 98.7% (663/672) and 98.9% (692/700) success rate in the AD and CN cohort, respectively. There were 9 missing tracts in the former and 8 in the latter. Method 2 generated 100% of tracts in both AD and CN cohorts. Implementation of Methods 1 and 2 on the ACPC aligned-EMCI cohort likewise showed high success rates of 96.2% (916/952) and 99.9% (951/952), respectively.

### Comparison of Diffusion Tensor Imaging Metrics Across Methodologies

Tables 2, 3 show the results of the paired *t*-tests conducted on the DTI metrics obtained from the two methodologies across all 8 white matter tracts. Table 2 compares the FA and MD obtained using Method 1 with those using Methods 2 and 3 applied on non-ACPC aligned images in the AD and CN cohorts, respectively. Table 3 also compares Methods 1 with 2 and 3 but applied on scans that have undergone the ACPC alignment and include scans from the EMCI cohort.

**TABLE 2:** Comparison of FA and MD derived by Method 1 against Methods 2 and 3 (non-ACPC aligned and co-registered) across white matter tracts in the **(A)** Alzheimer’s disease cohort and **(B)** cognitively normal cohort.

(A) AD cohort									

		Linear correlation		Significance of correlation		Mean difference		Paired *t*-test significance	
Tract		1 vs. 2	1 vs. 3	1 vs. 2	1 vs. 3	1 vs. 2	1 vs. 3	1 vs. 2	1 vs. 3
bCC	FA	0.150		0.308		–0.099		<0.001	
	MD	0.514		<0.001		0.0004		<0.001	
gCC	FA	0.226	0.494	0.127	<0.001	–0.144	–0.237	<0.001	<0.001
	MD	0.511	0.557	<0.001	<0.001	0.0003	0.0004	<0.001	<0.001
ATR	FA	0.416	0.378	0.004	0.009	–0.136	–0.136	<0.001	<0.001
	MD	0.502	0.281	<0.001	0.056	0.0005	0.0005	<0.001	<0.001
IFO	FA	0.469	0.474	<0.001	<0.001	–0.213	–0.208	<0.001	<0.001
	MD	0.421	0.406	0.003	0.005	0.0002	0.0002	<0.001	<0.001
ILF	FA	0.291	0.091	0.047	0.542	–0.236	–0.230	<0.001	<0.001
	MD	0.124	0.138	0.408	0.355	0.0002	0.0002	<0.001	<0.001
PLIC	FA	0.145		0.330		–0.086		<0.001	
	MD	0.054		0.716		0.0002		<0.001	
PTR	FA	0.352		0.015		–0.100		<0.001	
	MD	0.298		0.042		0.0001		<0.001	
UF	FA	–0.045	0.237	0.762	0.109	–0.202	–0.207	<0.001	<0.001
	MD	0.263	0.396	0.074	0.006	0.0008	0.0005	<0.001	<0.001

**(B) CN cohort**									

		**Linear correlation**		**Significance of correlation**		**Mean difference**		**Paired *t*-test significance**	
**Tract**		**1 vs. 2**	**1 vs. 3**	**1 vs. 2**	**1 vs. 3**	**1 vs. 2**	**1 vs. 3**	**1 vs. 2**	**1 vs. 3**

bCC	FA	0.415		0.003		–0.066		<0.001	
	MD	0.502		<0.001		0.0003		<0.001	
gCC	FA	0.354	0.602	0.012	<0.001	–0.124	–0.234	<0.001	<0.001
	MD	0.520	0.459	<0.001	<0.001	0.0002	0.0003	<0.001	<0.001
ATR	FA	0.340	0.301	0.017	0.036	–0.127	–0.128	<0.001	<0.001
	MD	0.481	0.545	<0.001	<0.001	0.0004	0.0004	<0.001	<0.001
IFO	FA	0.547	0.505	<0.001	<0.001	–0.217	–0.213	<0.001	<0.001
	MD	0.607	0.554	<0.001	<0.001	0.0001	0.0001	<0.001	<0.001
ILF	FA	0.427	0.290	0.002	0.041	–0.235	–0.227	<0.001	<0.001
	MD	0.571	0.532	<0.001	<0.001	0.0001	0.0001	<0.001	<0.001
PLIC	FA	–0.022		0.879		–0.058		<0.001	
	MD	0.182		0.205		0.0001		<0.001	
PTR	FA	0.174		0.232		–0.086		<0.001	
	MD	0.479		<0.001		0.0000		0.296	
UF	FA	–0.086	0.445	0.554	0.001	–0.205	–0.206	<0.001	<0.001
	MD	0.154	0.389	0.286	0.005	0.0005	0.0004	<0.001	<0.001

*A negative mean difference indicates that FA/MD derived by Method 1 is higher than that of Methods 2 or 3. All mean difference of MD is in mm^2^/s.*

**TABLE 3:** Comparison of FA and MD derived by Method 1 against Methods 2 and 3 (ACPC aligned and co-registered) across white matter tracts in the **(A)** Alzheimer’s disease cohort, **(B)** cognitively normal cohort, and **(C)** early mild cognitive impairment cohort.

(A) AD cohort									

		Linear correlation		Significance of correlation		Mean difference		Paired *t*-test significance	
Tract		1 vs. 2	1 vs. 3	1 vs. 2	1 vs. 3	1 vs. 2	1 vs. 3	1 vs. 2	1 vs. 3
bCC	FA	0.271		0.062		–0.073		<0.001	
	MD	0.506		<0.001		0.0004		<0.001	
gCC	FA	0.204	0.509	0.163	<0.001	–0.127	–0.244	<0.001	<0.001
	MD	0.686	0.561	<0.001	<0.001	0.0003	0.0004	<0.001	<0.001
ATR	FA	0.325	0.422	0.024	0.003	–0.131	–0.133	<0.001	<0.001
	MD	0.715	0.775	<0.001	<0.001	0.0005	0.0005	<0.001	<0.001
IFO	FA	0.550	0.537	<0.001	<0.001	–0.212	–0.215	<0.001	<0.001
	MD	0.307	0.323	0.034	0.025	0.0002	0.0002	<0.001	<0.001
ILF	FA	0.122	0.173	0.408	0.241	–0.235	–0.233	<0.001	<0.001
	MD	0.302	0.311	0.037	0.032	0.0002	0.0002	<0.001	<0.001
PLIC	FA	0.104		0.484		–0.074		<0.001	
	MD	0.312		0.031		0.0002		<0.001	
PTR	FA	0.337		0.019		–0.086		<0.001	
	MD	0.406		0.004		0.0000		0.060	
UF	FA	0.115	0.446	0.436	0.001	–0.202	–0.210	<0.001	<0.001
	MD	0.281	0.392	0.053	0.006	0.0009	0.0005	<0.001	<0.001

**(B) CN cohort**									

		**Linear correlation**		**Significance of correlation**		**Mean difference**		**Paired *t*-test significance**	
**Tract**		**1 vs. 2**	**1 vs. 3**	**1 vs. 2**	**1 vs. 3**	**1 vs. 2**	**1 vs. 3**	**1 vs. 2**	**1 vs. 3**

bCC	FA	0.302		0.033		–0.055		<0.001	
	MD	0.503		<0.001		0.0004		<0.001	
gCC	FA	0.268	0.426	0.062	0.002	–0.109	–0.245	<0.001	<0.001
	MD	0.556	0.510	<0.001	<0.001	0.0002	0.0003	<0.001	<0.001
ATR	FA	0.488	0.473	<0.001	<0.001	–0.116	–0.120	<0.001	<0.001
	MD	0.688	0.672	<0.001	<0.001	0.0004	0.0004	<0.001	<0.001
IFO	FA	0.548	0.424	<0.001	0.002	–0.213	–0.215	<0.001	<0.001
	MD	0.730	0.683	<0.001	<0.001	0.0001	0.0002	<0.001	<0.001
ILF	FA	0.331	0.317	0.019	0.025	–0.234	–0.233	<0.001	<0.001
	MD	0.583	0.618	<0.001	<0.001	0.0001	0.0001	<0.001	<0.001
PLIC	FA	–0.191		0.184		–0.047		<0.001	
	MD	0.181		0.208		0.0001		<0.001	
PTR	FA	0.114		0.429		–0.069		<0.001	
	MD	0.437		0.002		0.0000		0.449	
UF	FA	–0.088	0.479	0.545	<0.001	–0.199	–0.209	<0.001	<0.001
	MD	0.123	0.422	0.394	0.002	0.0005	0.0004	<0.001	<0.001

**(C) EMCI cohort**									

		**Linear correlation**		**Significance of correlation**		**Mean difference**		**Paired *t*-test significance**	
**Tract**		**1 vs. 2**	**1 vs. 3**	**1 vs. 2**	**1 vs. 3**	**1 vs. 2**	**1 vs. 3**	**1 vs. 2**	**1 vs. 3**

bCC	FA	0.334		0.006		–0.048		<0.001	
	MD	0.436		<0.001		0.0004		<0.001	
gCC	FA	0.414	0.553	<0.001	<0.001	–0.106	–0.245	<0.001	<0.001
	MD	0.420	0.440	<0.001	<0.001	0.0002	0.0003	<0.001	<0.001
ATR	FA	0.460	0.483	<0.001	<0.001	–0.127	–0.129	<0.001	<0.001
	MD	0.227	0.535	0.069	<0.001	0.0005	0.0005	<0.001	<0.001
IFO	FA	0.628	0.597	<0.001	<0.001	–0.208	–0.209	<0.001	<0.001
	MD	0.572	0.523	<0.001	<0.001	0.0002	0.0002	<0.001	<0.001
ILF	FA	0.502	0.558	<0.001	<0.001	–0.231	–0.230	<0.001	<0.001
	MD	0.531	0.564	<0.001	<0.001	0.0001	0.0001	<0.001	<0.001
PLIC	FA	0.114		0.361		–0.275		<0.001	
	MD	0.360		0.003		0.0001		<0.001	
PTR	FA	0.438		<0.001		–0.063		<0.001	
	MD	0.570		<0.001		0.0000		0.008	
UF	FA	0.161	0.562	0.197	<0.001	–0.193	–0.205	<0.001	<0.001
	MD	0.398	0.580	0.001	<0.001	0.0006	0.0004	<0.001	<0.001

*A negative mean difference indicates that FA/MD derived by Method 1 is higher than that of Methods 2 or 3. All mean difference of MD is in mm^2^/s.*

Non-ACPC aligned Method 1 was not well correlated to Methods 2 and 3 (Table 2). FA and MD from Method 1 were significantly different (*p* < 0.001) from Methods 2 and 3 for all tracts in AD and CN cohorts, except for PTR MD in CN. After ACPC alignment and co-registration, Method 1 was significantly different (*p* < 0.001) from Methods 2 and 3 for all tracts in AD, CN, and EMCI cohorts, except for PTR MD in AD and CN (Table 3).

**FIGURE 1:**
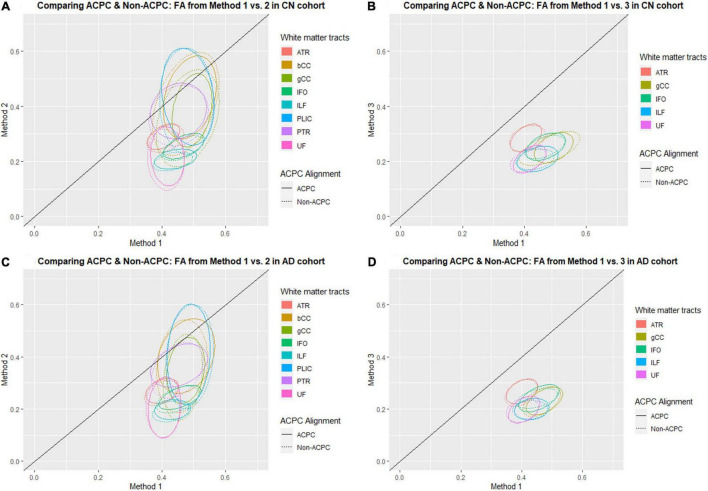
Distribution of scatter plots of FA obtained using Methods 2 and 3 against Method 1, comparing non-ACPC aligned and co-registered with ACPC aligned white matter tracts across cognitively normal (CN) and Alzheimer’s Disease (AD) cohorts. The ellipses assume a multivariate normal distribution with the mean at the center and area of the ellipse representing 95% confidence level. gCC, genu of the corpus callosum; bCC, body of the corpus callosum; ATR, anterior thalamic radiation; IFO, inferior fronto-occipital fasciculus; ILF, inferior longitudinal fasciculus; PLIC, posterior limb of the internal capsule; PTR, posterior thalamic radiation; UF, uncinate fasciculus. **(A)** Comparing ACPC and Non-ACPC: FA from Method 1 vs. 2 in CN cohort. **(B)** Comparing ACPC and Non-ACPC: FA from Method 1 vs. 3 in CN cohort. **(C)** Comparing ACPC and Non-ACPC: FA from Method 1 vs. 2 in AD cohort. **(D)** Comparing ACPC and Non-ACPC: FA from Method 1 vs. 3 in AD cohort.

**FIGURE 2:**
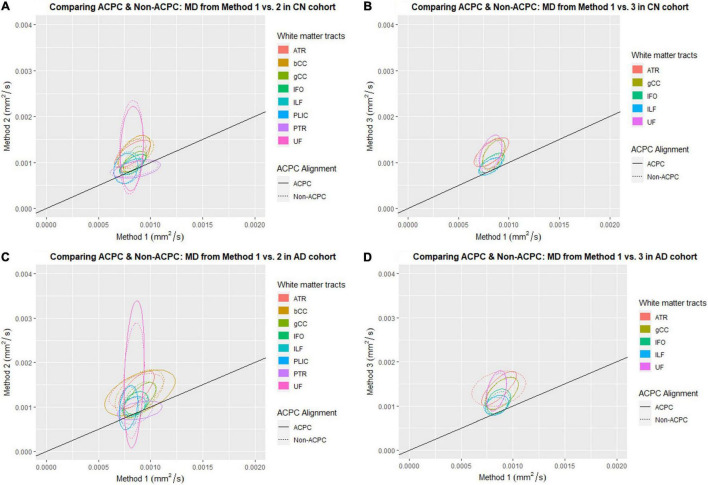
Distribution of scatter plots of MD obtained using Methods 2 and 3 against Method 1, comparing non-ACPC aligned and co-registered with ACPC aligned white matter tracts across cognitively normal (CN) and Alzheimer’s Disease (AD) cohorts. The ellipses assume a multivariate normal distribution with the mean at the center and area of the ellipse representing 95% confidence level. gCC, genu of the corpus callosum; bCC, body of the corpus callosum; ATR, anterior thalamic radiation; IFO, inferior fronto-occipital fasciculus; ILF, inferior longitudinal fasciculus; PLIC, posterior limb of the internal capsule; PTR, posterior thalamic radiation; UF, uncinate fasciculus. **(A)** Comparing ACPC and Non-ACPC: MD from Method 1 vs. 2 in CN cohort. **(B)** Comparing ACPC and Non-ACPC: MD from Method 1 vs. 3 in CN cohort. **(C)** Comparing ACPC and Non-ACPC: MD from Method 1 vs. 2 in AD cohort. **(D)** Comparing ACPC and Non-ACPC: MD from Method 1 vs. 3 in AD cohort.

**FIGURE 3:**
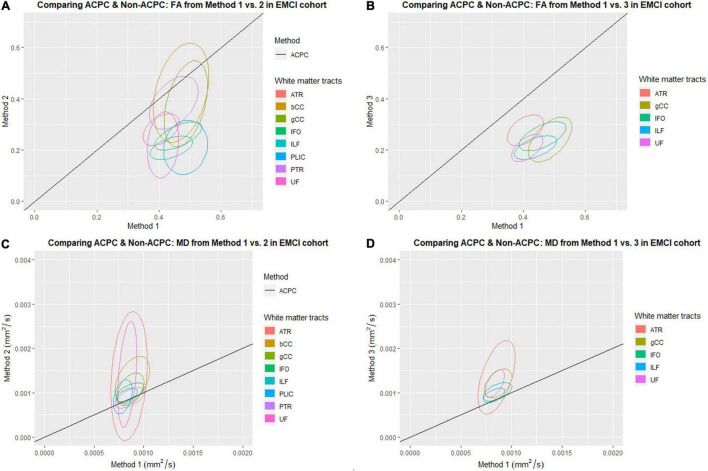
Distribution of scatter plots of FA and MD obtained using Methods 2 and 3 against Method 1 (ACPC aligned and co-registered) across white matter tracts in the early mild cognitive impairment (EMCI) cohort. The ellipses assume a multivariate normal distribution with the mean at the center and area of the ellipse representing 95% confidence level. gCC, genu of the corpus callosum; bCC, body of the corpus callosum; ATR, anterior thalamic radiation; IFO, inferior fronto-occipital fasciculus; ILF, inferior longitudinal fasciculus; PLIC, posterior limb of the internal capsule; PTR, posterior thalamic radiation; UF, uncinate fasciculus. **(A)** Comparing ACPC and Non-ACPC: FA from Method 1 vs. 2 in EMCI cohort. **(B)** Comparing ACPC and Non-ACPC: FA from Method 1 vs. 3 in EMCI cohort. **(C)** Comparing ACPC and Non-ACPC: MD from Method 1 vs. 2 in EMCI cohort. **(D)** Comparing ACPC and Non-ACPC: MD from Method 1 vs. 3 in EMCI cohort.

Figure 1 compares the FA of ACPC aligned images and non-ACPC aligned images obtained using Methods 2 and 3 against Method 1 in the 8 white matter tracts across CN and AD cohorts. Figure 2 compares the MD of ACPC aligned images and non-ACPC aligned images obtained using Methods 2 and 3 against Method 1. Figure 3 compares both the FA and MD of ACPC aligned images using Methods 2 and 3 against Method 1 in the EMCI cohort. Non-ACPC aligned images in the EMCI cohort were not compared here because the results were similar to AD and CN images. The findings here seem to generally agree with those from the paired *t*-tests.

FA and MD scatter plots demonstrated poor agreement between Method 1 vs. 2 and Method 1 vs. 3 across all tracts in AD and CN (Figures 1, 2). This was not improved even with ACPC alignment and co-registration of images. These trends are also present with the addition of a cohort with an intermediate severity of disease process (i.e., EMCI).

Across the paired *t*-tests in Tables 2, 3 and scatter plots in Figures 1–3, FA and MD obtained using Methods 1, 2, and 3 show poor agreement and consistency across the CN, AD, and EMCI cohorts. This is evidenced by the low linear correlation coefficients and relatively large mean differences in the metrics obtained across all tracts and cohorts as well as the scatterplots showing a large deviation from the 45-degree diagonal line. This demonstrates that the type of standard atlases used in Method 2 (i.e., an alternative atlas was also tested using Method 3) did not meaningfully improve the agreement. Additionally, comparing across the AD and CN cohorts also showed no changes in agreements. Implementing the ACPC alignment across all scans improved the agreements across all tracts only marginally. Notably, the inter-methodological differences were greater than the differences due to the application of technical considerations. This confirmed the fact that there were external confounding factors impacting the methodologies which rendered them incomparable.

### White Matter Pattern Changes in Cognitively Normal vs. Early Mild Cognitive Impairment vs. Alzheimer’s Disease Cohorts

Despite the lack of agreement, Methods 1, 2, and 3 showed general trends that illustrate and reaffirm the presence of different white matter pattern changes when comparing across the AD, EMCI, and CN cohorts with varying degrees of neurodegeneration. Figures 1–3 show that there was greater variability for white matter structures adjacent or near to the ventricles such as the bCC and gCC as well as multidirectional tracts like the PLIC and UF. The difference, however, is that Method 1 reports white matter tracts having generally higher FA and lower MD values, implying a more preserved neural structure, compared to the other two methods for each cohort tested.

### Effect on White Matter Pattern Changes With Increasing Ventriculomegaly

**FIGURE 4:**
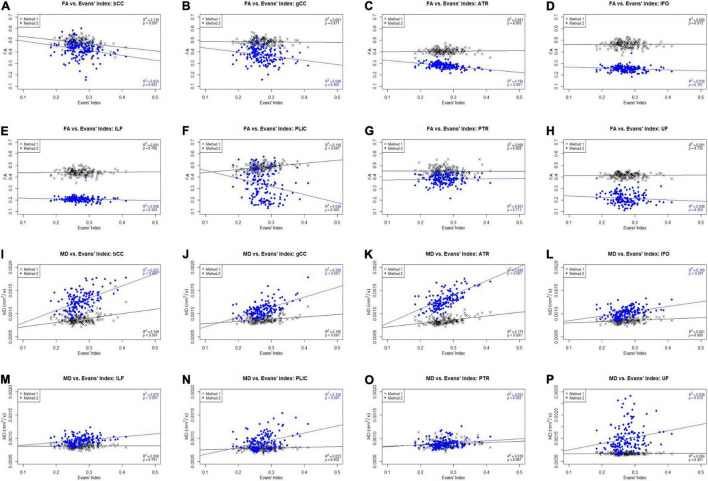
Scatter plots of FA and MD against Evans’ index (EI) across white matter tracts for CN, AD, and EMCI cohorts combined. gCC, genu of the corpus callosum; bCC, body of the corpus callosum; ATR, anterior thalamic radiation; IFO, inferior fronto-occipital fasciculus; ILF, inferior longitudinal fasciculus; PLIC, posterior limb of the internal capsule; PTR, posterior thalamic radiation; UF, uncinate fasciculus. **(A)** FA vs. Evans’ Index: bCC. **(B)** FA vs. Evans’ Index: gCC. **(C)** FA vs. Evans’ Index: ATR. **(D)** FA vs. Evans’ Index: IFO. **(E)** FA vs. Evans’ Index: ILF. **(F)** FA vs. Evans’ Index: PLIC. **(G)** FA vs. Evans’ Index: PTR. **(H)** FA vs. Evans’ Index: UF. **(I)** MD vs. Evans’ Index: bCC. **(J)** MD vs. Evans’ Index: gCC. **(K)** MD vs. Evans’ Index: ATR. **(L)** MD vs. Evans’ Index: IFO. **(M)** MD vs. Evans’ Index: ILF. **(N)** MD vs. Evans’ Index: PLIC. **(O)** MD vs. Evans’ Index: PTR. **(P)** MD vs. Evans’ Index: UF.

Figure 4 shows the scatter plots of FA and MD obtained from both Methods 1 and 2 plotted against the Evans’ index (EI) for all 8 white matter tracts across all 3 cohorts of AD, EMCI, and CN combined. Only the ACPC aligned images are used in this analysis due to its superior reliability and reproducibility as previously shown. From the figures, we observed differing patterns of correlation with EI when using Method 1 compared to Method 2. From metrics obtained using Method 2, as EI increased (implying increasing ventriculomegaly) there was a significant decrease in FA which is consistent with decreased white matter integrity. By contrast, metrics obtained using Method 1, showed that with increasing EI, there was a significant increase in FA seen in the PLIC which is consistent with patterns of stretch or compression. Both methods showed significant increases in MD with increasing EI, suggesting an increase in global, multi-directional white matter disruption. Method 1, however, showed less variability and was more specific to changes in white matter tracts near to the ventricles.

### Correlation to Diffusion Tensor Imaging Profiles of White Matter Tracts

**FIGURE 5:**
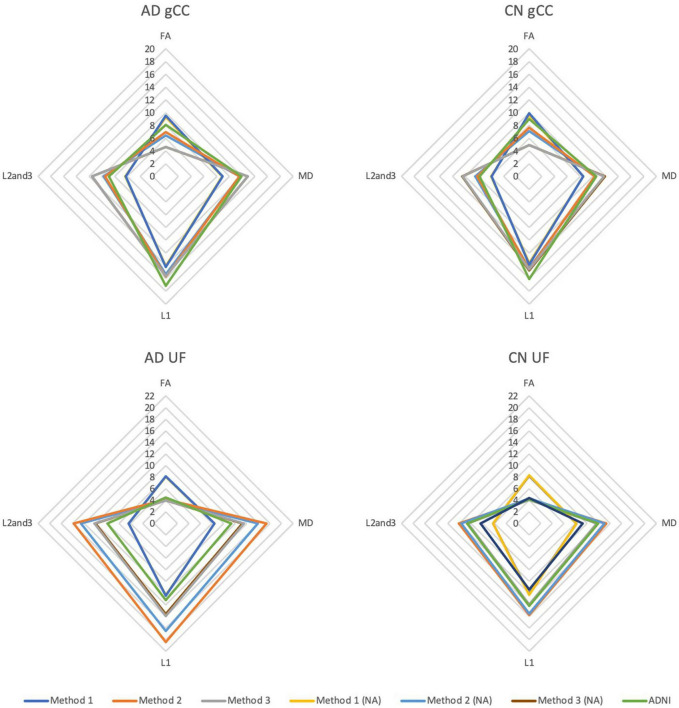
DTI radar graph profiles for comparison of methods with and without ACPC alignment and co-registration in Alzheimer’s disease (AD) and cognitively normal (CN) cohorts. DTI metrics from the ADNI data archive are included for reference. FA values are presented as x20 for illustration; MD, L1, L2, and 3 values are presented as ×10^4^. gCC, genu of the corpus callosum; UF, uncinate fasciculus; NA, no ACPC alignment.

**FIGURE 6:**
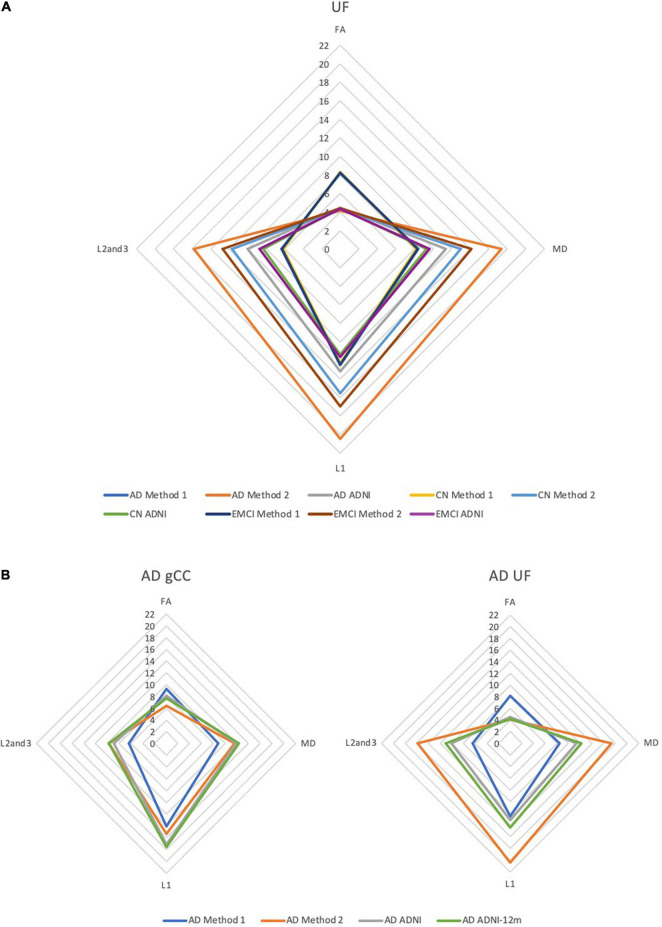
DTI radar graph profiles for comparison of differences in methods vs. **(A)** cohort differences and **(B)** changes in the AD cohort after 12 months. DTI metrics from the ADNI data archive are included for reference. FA values are presented as ×20 for illustration; MD, L1, L2, and 3 values are presented as ×10^4^. AD, Alzheimer’s disease; CN, cognitively normal; EMCI, early mild cognitive impairment; gCC, genu of the corpus callosum; UF, uncinate fasciculus.

**FIGURE 7:**
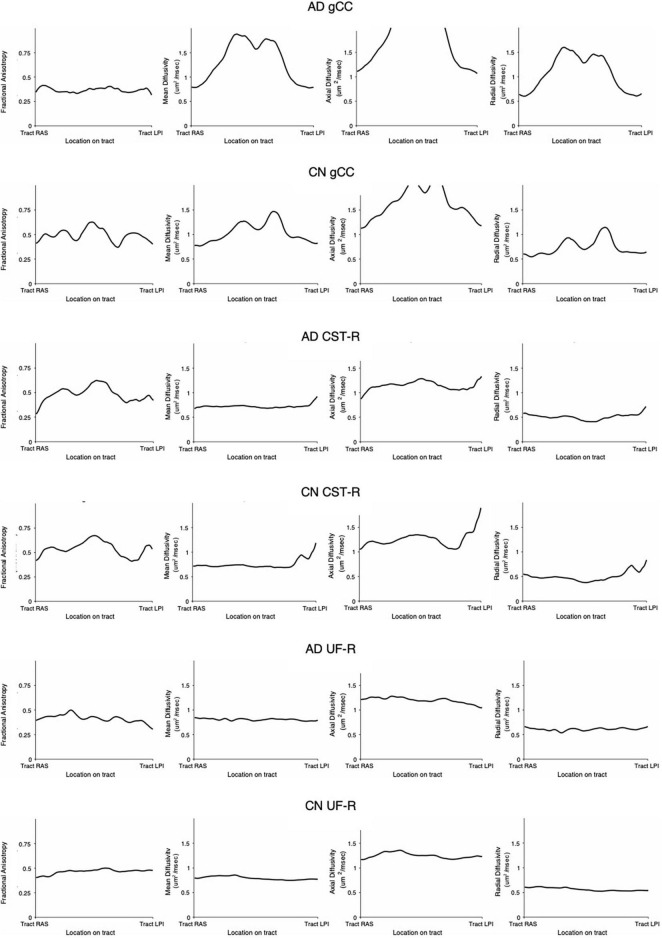
White matter tract profiles demonstrating variability along tracts. AD, Alzheimer’s disease; CN, cognitively normal; gCC, genu of the corpus callosum; CST-R, corticospinal tract (right); UF-R, uncinate fasciculus (right).

DTI profiles for the gCC and the UF tracts were selected to illustrate differences between the methods and between cohorts. Figure 5 shows that the difference in DTI metrics generated by Methods 1, 2, and 3 can be distinguished with DTI profiles. Method 1 consistently produced the lowest MD, L1 and L2, and 3 values, compared to Methods 2 and 3. Profiles for DTI metrics before and after ACPC alignment were nearly visually indistinguishable for Methods 1 and 2. Failure to correct for ACPC alignment did not influence the results as much as the variation produced by the different methods. DTI profiles demonstrated cohort differences between AD, CN, and EMCI, across the spectrum of disease, but inter-methodological differences were larger than inter-cohort differences (Figure 6A). Likewise, change in DTI morphology in the AD cohort after 12 months was not as pronounced as inter-methodological differences (Figure 6B). White matter tract profiles in Figure 7 show the variability of DTI metrics along different white matter tracts.

## Discussion

In this paper, we demonstrated that it was possible to reliably develop and refine an SOP for a pseudo atlas-based semi-automated tractography DTI analysis method in the presence of confounders comprising aging, neurodegenerative disease, and ventricular enlargement. However, the absolute values of the DTI metrics generated by this novel methodology did not align well with those generated by standardized atlas-based DTI analyses, despite implementing a differential of algorithmic modifications. Regardless, we managed to show that the inter-methodological differences between DTI metrics obtained from Method 1 and 2 were greater than the effects of implementing the algorithmic modifications. Whilst this suggests that DTI output metrics from differing methodologies cannot be directly compared for statistical analysis, we also showed that DTI methodologies were differentially impacted upon by confounders affecting structural brain or ventricular changes. In the presence of such considerations, we found that there was no true “gold-standard” but rather, the differing methodologies were sensitive to differing significant findings on a spectrum from contiguous to non-contiguous changes, in ways that were both complementary to each other and consistent with differences between such cohorts as reported in published literature. Nevertheless, by creating the DTI profiles from metrics generated by the methodologies, we showed that, despite differing DTI values, the morphology of DTI changes was consistent across DTI analysis methods.

### A Cohort-Specific Pseudo Atlas-Based Semi-Automated Tractography Method vs. Standardized Atlas-Based Diffusion Tensor Imaging Analysis

In our study, we found that a novel pseudo atlas-based semi-automated tractography DTI analysis method (Method 1) was reliable and reproducible. This was evidenced by the high success rate of generating white matter tracts across both AD and CN sub cohorts. Upon implementing the ACPC alignment (one modification to the algorithm), the number of missing tracts decreased from 25 to 9 tracts in the AD cohort and 22–8 tracts in the CN cohorts. This showed that the intracohort variability in image orientation could be a main contributor to the missing tracts and that this refinement improved the reliability and reproducibility of the methodology.

Surprisingly, we could not show that the actual DTI metrics generated from the pseudo atlas-based semi-automated tractography DTI analysis method (Method 1) were exactly comparable to the standardized atlas-based DTI analysis (Methods 2). This was despite implementing the both modifications to the algorithm including an alternative published and verified atlas (Method 3) and applying the ACPC alignment. From observing the scatter plots in Figures 1–3 we noted that this disagreement can be attributed to Method 1 reporting white matter tracts as having generally higher FA and lower MD values compared to those obtained via Methods 2 and 3 across AD, CN, and EMCI cohorts. However, as this finding was consistent across the varying spectrums of disease and aging, i.e., in AD (neurodegenerative), EMCI (mild neurodegenerative) and CN (aging) cohorts, it suggests that a cohort-specific white matter template (the pseudo-atlas) as employed in Method 1 was more sensitive to generating white matter tracts in the presence of confounders compared to Methods 2 and 3. This showed that while Method 1 was internally consistent across cohorts, DTI output measures may not be directly comparable to DTI measures from Methods 2 and 3 for purposes of statistical analysis.

### The Effect of Ventriculomegaly on the Degree of Patterns of White Matter Change

From Figure 4, we noted that with increasing EI, signifying an increasing degree of ventriculomegaly, Method 1 showed that PLIC had a significant increase in FA, Method 2 showed a significant decrease in FA across all tracts and both Methods 1 and 2 showed significant increases in MD across all tracts. An increase in FA is consistent with tracts under stretch or compression while a decrease in FA is consistent with decreased white matter integrity. Conversely, a rise in MD across all tracts suggested an increase in global, multidirectional white matter disruption. These patterns of white matter injury in the setting of ventriculomegaly are consistent with findings reported in the literature (Keong et al., 2017). Whilst the FA changes in PLIC reflected in Methods 1 and 2 appear to be contradictory, such conflict is consistent with DTI findings of previous work in hydrocephalus where it was shown that FA can increase and decrease within the same context, depending on the reversibility of white matter injury (Assaf et al., 2006; Hattori et al., 2011; Kanno et al., 2011; Ben-Sira et al., 2015; Keong et al., 2017; Tan et al., 2018). This represents an important fallacy of interpreting DTI changes based solely on global measures, such as FA or MD alone. In particular, FA is highly dependent upon relative changes in diffusivity measures; it can be driven to higher or lower values based on predominant changes in axial diffusivity over radial diffusivity and vice versa. In this study, we found that the different patterns that were reflected in both methods could be interpreted as complementary to each other. For example, Method 2 may have detected the reduced white matter integrity and hence decreased FA, whereas Method 1 detected the compressive mechanism of injury and hence increased FA. Method 1, however, showed less variability and was more specific to changes in white matter tracts nearer to the ventricles (i.e., bCC, gCC), when compared to Method 2. These known DTI conflicts impacting upon the transparency and consistency of interpretation of DTI results across literature would benefit from the application of a more standardized common taxonomy; this is an approach we have proposed elsewhere (Keong et al., 2017, manuscript in submission).

### Correlation to Diffusion Tensor Imaging Profiles

The DTI profiles in Figures 5, 6 align with the above findings. We showed that Method 1 reports more preserved white matter profiles in comparison to Methods 2 and 3 across AD, CN and EMCI cohorts. This supports the suggestion that a cohort-specific template (the pseudo-atlas) was more sensitive to demonstrating white matter integrity in the presence of confounders due to aging and neurodegenerative disease. Additionally, we found evidence that ACPC alignment did not significantly affect the morphology of DTI profiles generated and that inter-methodological differences were indeed larger than inter-cohort differences. These cohorts include the spectrum of Alzheimer’s disease, from CN to EMCI and finally to AD. Inter-methodological differences were similar to or greater than changes in DTI profiles in the AD cohort after 12 months.

Despite the variability of DTI values along the tracts (Figure 7) as well as between methodologies (as seen in DTI profiles), the morphology of the DTI profile still remains consistent across cohorts and aligns well with published literature. This lack of comparability in DTI analysis methodologies and variability, ultimately supports the use of DTI profiles in the analysis of DTI metrics.

### Strengths and Weakness of Differing Diffusion Tensor Imaging Methodologies

As we have previously discussed, the success rate in generating white matter tracts is marginally higher (after the ACPC alignment) for Method 2 compared to Method 1. This is likely because our use of a single subject pseudo atlas restricted the automated tractography, rendering it more selective in its ability to generate the white matter tracts. This can be seen as an advantage to Method 1 as its selectiveness may reduce the likelihood of generating spurious tracts and thus erroneous data. The use of Method 2 incorporated the use of validated atlases which have been derived from group-averaging from a sample cohort. This may also be perceived as providing this option with a technical advantage over Method 1, which used a single subject selected from the dataset to generate the pseudo-atlas. However, as we have discovered, the use of a pseudo-atlas may equally be argued to be advantageous as it promotes a template that is more representative of the cohorts compared to the standardized atlases used in Methods 2 and 3. Our study has shown that this resulted in Method 1 (the pseudo-atlas) being more sensitive than standardized atlas-based DTI analyses, in characterizing changes in the model of white matter at-risk due to pathophysiological processes of distortion and disease. In terms of processing, Method 1 required a much longer time to produce the DTI data compared to Methods 2 or 3. This was for two main reasons. The first was that the white matter tracts had to be manually generated in the pseudo-atlas template prior to performing the tractography. This process could be lengthy and required individuals with a working knowledge of neuroanatomy to perform. Additionally, there could be inherent subjectivity when it came to generating the tracts because it was difficult to determine if there were missing “strands” of white matter or conversely, if spurious “strands” were being generated. Secondly, the tractography itself of Method 1 also required a long time, which required approximately 3 h to generate a single tract from a single DWI. In contrast, Methods 2 and 3 did not require manual generation of the white matter tracts as it utilized readily available atlases compiled and verified by other groups. In terms of processing speed, Method 2 was about 50 times faster than Method 1, requiring 3 h to generate a single tract from a cohort of 50 DWIs.

### Study Limitations

DTI has a low specificity and is generally due to its low signal to noise ratio (Ranzenberger and Snyder, 2022). As a result, the imaging quality may be poor, and artifacts become a problem. Additionally, the DTI metrics are highly dependent on the size of the voxel during analysis. A single voxel may contain multi-directional structures which can result in inaccurate DTI measurements. Ideally, a single voxel should be small enough that it encompasses a single white matter bundle, taking a point measurement of DTI metrics. Therefore, the inter-methodological differences found in our paper could be in part be attributed to its low signal to noise ratio.

In this study, we only considered two disease cohorts (AD and EMCI) and healthy controls (CN). The full ADNI dataset included other cohorts along the disease spectrum, such as the late mild cognitive impairment (LMCI) and significant memory concern (SMC) cohorts. With further analysis it may have been possible that one of the three methods chosen would have emerged as the predominantly reliable and reproducible method of DTI analysis, with findings entirely consistent with literature. In addition, despite widespread use of ROI methodologies in literature, manual specifications and semi-automated tractography may be considered less reliable than fully automated white matter analytical approaches. Nevertheless, due to their ubiquity, results from this study would be easily translated to other settings involving DTI analysis at the clinical-research interface.

In Method 1, a randomly selected image from each study cohort was used as an atlas. This may have potentially introduced bias as we could not be certain that the selected images were adequately representative of the entire cohort. However, the selected images were inspected for abnormal or outrightly distinctive features that could render them significantly different from other images within the cohort. Future work might include creating a more representative atlas by generating a grouped average of multiple images from the cohort.

We also recognize that previous studies that have utilized a representative cohort-specific subject-based approach to DTI analysis have explored and demonstrated its limitations. For example, Keihaninejad et al. (2012) compared different methods of registration schemes for the use of TBSS for DTI analysis (Standard, Most-Representative-Subject, Study-Specific-Template, and Group-wise) in terms of their performance in reducing misalignment within the context of Alzheimer’s disease and large deformations due to atrophy. They found that the approaches studied all showed false-positive error in evaluation of specificity, likely due to variations in levels of white matter atrophy and ventricular size. However, it was possible to improve the performance of aligning DTI data using a group-wise average atlas approach (Keihaninejad et al., 2012). The degree of confounders such as white matter atrophy and ventriculomegaly can be highly variable between patients; it could therefore be equally argued that, in certain cohorts such as ours, the use of a Most-Representative Subject approach may still be more advantageous, since we would expect the white matter pattern changes to affect similar “at-risk” locations within the same disease process but group-wise averaging may introduce further unintended distortions to the template of the “at-risk atlas” of disease. Nevertheless, our study showed that even in the absence of confounders such as atrophy and ventriculomegaly as in the CN cohort, and despite implementation of the algorithmic modifications. There is still a poor agreement between methodologies, which supports our conclusion that no true “gold-standard” DTI methodology exists without limitations for all possible disease datasets of interest.

It is also important to note that the use of Evans’ Index as a marker for ventriculomegaly is imperfect because it is dependent on the inter-rater reliability at measuring the maximal width of the frontal horns and the internal diameter of the skull. These measurements are also highly dependent on the chosen slice and location at which the markers are placed. In addition, the orientation of each image has a large influence on the slices and thus the measurements. Although this effect is mitigated by alignment of the commissures, such technical considerations should be addressed and optimized by each rater, prior to its application as a biomarker for ventricular enlargement across a range of datasets.

### Future Work

We plan to expand our analyses using both DTI methodologies, to include other cohorts of interest along the spectrum of AD and other neurodegenerative diseases. We also aim to use other anatomical segmentation methods to examine macro-structural features of white matter, such as its volume and thickness, as well as to create topological maps of adjacent surfaces, in order to augment the interpretation of the morphology of white matter changes, as described by DTI profiles. In the context of ventriculomegaly, we plan to utilize complementary biomarkers for both 2-dimensional and 3-directional measures in specific groups that possess significant ventriculomegaly such as cohorts with NPH. Finally, we aim to further expand the concept of DTI profiles as an invaluable tool toward boosting our capacity to compare the interpretation of DTI findings across methodologies which are not directly comparable using conventional statistical methods.

## Conclusion

In this study, we found that there was no true gold-standard for DTI methodologies or atlases. It was possible to create a pseudo-atlas that was cohort-specific for immediate study use. Whilst there was no congruence between absolute values from DTI metrics, differing DTI methodologies were still valid but must be appreciated to be variably sensitive to different changes within white matter injury occurring concurrently. When such changes were found to exist in the same dataset, the use of differing methods were complementary in elucidating the characterization of such DTI changes. We found that, despite such algorithmic modifications, the use of DTI profiles, a methodology of distilling the complexity of DTI changes to their most simplistic, graphical forms, confirmed the morphology of white matter injury as described by DTI metrics, remained consistent. By combining both atlas and pseudo-atlas based methodologies with DTI profiles, it was possible to navigate past such challenges to describe white matter injury changes in the context of confounders, such as neurodegenerative disease and ventricular enlargement, with transparency and consistency.

## Data Availability Statement

The data will be made available but is subject to approval from the ADNI (Alzheimer’s Disease Neuroimaging Initiative) as they own the scans that the data were derived from.

## Ethics Statement

Data used in preparation of this article were obtained from the ADNI database (adni.loni.usc.edu). Written informed consent was obtained from all ADNI subjects, and participating sites in the ADNI study received approval from their respective governing Institutional Review Boards.

## Author Contributions

CK, NK, and CL conceptualized and designed the study methodology, wrote the manuscript, and contributed to the analysis and interpretation of study data. CK, NK, CL, and TA contributed to data collection and validation. All authors contributed to the article and approved the submitted version.

## Conflict of Interest

The authors declare that the research was conducted in the absence of any commercial or financial relationships that could be construed as a potential conflict of interest.

## Publisher’s Note

All claims expressed in this article are solely those of the authors and do not necessarily represent those of their affiliated organizations, or those of the publisher, the editors and the reviewers. Any product that may be evaluated in this article, or claim that may be made by its manufacturer, is not guaranteed or endorsed by the publisher.

## Footnotes

1
adni.loni.usc.edu
